# Prevalence and characterization of tinnitus in patients with mental disorders in the context of primary health care

**DOI:** 10.1590/2317-1782/e20250247en

**Published:** 2026-04-07

**Authors:** Rubens Jonatha dos Santos Ferreira, Camila Nascimento Monteiro, Daiane Aparecida Dias, Marine Raquel Diniz da Rosa, Maria Fernanda Capoani Garcia Mondelli

**Affiliations:** 1 Programa de Pós-graduação em Fonoaudiologia, Faculdade de Odontologia de Bauru – FOB, Universidade de São Paulo – USP - Bauru (SP), Brasil.; 2 Departamento de Saúde Populacional, Hospital Sírio-Libanês - São Paulo (SP), Brasil.; 3 Programa Associado de Pós-graduação em Fonoaudiologia, Universidade Federal da Paraíba – UFPB - João Pessoa (PB), Brasil.

**Keywords:** Tinnitus, Mental Health, Primary Health Care, Mental Disorders, Comprehensive Health Care Practice

## Abstract

**Purpose:**

To estimate the prevalence and characterize tinnitus in patients with mental disorders.

**Methods:**

Observational study conducted with patients enrolled in the mental health care pathway of a primary health care service within the supplementary system. The Tinnitus Handicap Inventory and the Visual Analog Scale were used to assess tinnitus, while the Depression Anxiety and Stress Scale was applied to evaluate psychobehavioral aspects.

**Results:**

Tinnitus was reported by nearly half of the study population. Stress was the most reported triggering factor, and higher scores of anxiety, depression, and stress were observed among participants with tinnitus. Most participants presented moderate discomfort, and overall burden ranged from negligible to mild.

**Conclusion:**

A high prevalence of tinnitus with moderate to severe discomfort was observed in participants with mental disorders. An association was found between tinnitus and severe symptoms of anxiety and stress, underscoring the importance of multidisciplinary management for this population.

## INTRODUCTION

Tinnitus is a symptom that is currently present in one out of adults, a number that is being increased by the triggering of the symptom as a consequence of COVID-19^(1,2)^. Analyses^(3)^ based on human behavior show models that emphasize an influential relationship between the cognitive response to tinnitus and the manifestation and level of discomfort associated with the symptom.

From a physiological point of view, some theories^(4)^ indicate that the perception of tinnitus activates brain areas related to executive functions, such as: prefrontal lobe, parietal lobe, cingulate cortex, insula, thalamus, auditory cortex, parahippocampus, hippocampus and amygdala. This pattern is reinforced by brain imaging studies of people with tinnitus^(5)^. Thus, for an assertive clinical management of patients who report tinnitus and cognitive difficulties, an understanding of this relationship and its possible additive effects of anxiety, depression and somatic cognitive bias^(3)^ must be considered.

The association between tinnitus and emotional disorders is described by Rosa et al.^(6)^, however, without characterization of the precursor agent, since the literature describes that the person with tinnitus may present a greater suicidal tendency, depression and anxiety, in addition to the cyclical relationship between anxiety, stress, depression and tinnitus, and its negative impact on quality of life.

Furthermore, the persistence of the symptom throughout the day and its impact on social and daily life activities can influence the development of additive behavioral and mental health effects^(3)^. Since the person with tinnitus is exposed to situations of stress and social isolation, social phobia behaviors and related psychobehavioral aspects tend to aggravate the perception of the symptom in the relationship between the limbic system and cognitive circuitry, which again refers to a constant cycle between these variables^(7)^.

Due to its multifactorial and heterogeneous nature, there is still no common explanation for the onset of tinnitus; however, it is known that this symptom can be present in different clinics and levels of health care, including Primary Health Care (PHC)^(8)^. PHC is an important component of health services, as it comprises the population base and provides support for the development of different care strategies and care pathways that result in better practices for promotion, prevention and rehabilitation.

Mental health care pathways play a fundamental role in the organization, systematization and provision of services, in order to guarantee more qualified and humanized care for patients, through the articulation and integration of the various levels of health care^(9)^. The role of Speech Therapy in mental health care pathways ranges from activities that promote the (re)integration of individuals into the social environment through the improvement of communicative skills to strategies for minimizing behavioral disorders associated with voice, hearing and swallowing disorders, which can be aggravated as a result of psychiatric conditions, such as tinnitus management^(10)^.

Although the mutual influence between tinnitus, anxiety, depression, and stress is widely recognized, there remains a gap in characterizing, in detail, the degrees of discomfort and their implications for emotional and behavioral outcomes in mental health patients. Thus, the present study seeks to fill this gap, and its findings may support the improvement of clinical practices and the enhancement of interdisciplinary care strategies, especially regarding early screening, comprehensive approaches, and the articulation between the fields of mental health and hearing health. Therefore, this study is based on the hypothesis that patients with tinnitus present higher scores on psychobehavioral symptoms when compared to those without the symptom. In this scenario, the present study aims to estimate the prevalence and characterize tinnitus in patients with mental disorders.

## METHODS

This is an observational and cross-sectional study, carried out with patients from a mental health program in the supplementary primary health care sector. The project was approved by the Research Ethics Committee of the Sírio-Libanês Hospital under No. 71386823.9.0000.5461. All participants signed the Informed Consent Form.

The sample size calculation was based on the population of 235 patients who were part of the healthcare pathway between June 2023 and June 2024. Considering a standard deviation of 1.5 points, a significance level of 5%, and a test power of 95%, the sample was determined to have a minimum of 147 participants. The inclusion criteria were participants over 18 years of age, of both sexes, who were being monitored in a mental health care program and had one or more diagnoses of common mental disorders (anxiety - ICD F40/F41; depression - ICD F32/F33; and/or stress - ICD F43). These diagnoses were previously made by physicians, based on clinical criteria and results of the scales used in the standard assessment of the service: General Anxiety Disorder-7 (GAD-7) and Patient Health Questionnaire-9 (PHQ-9). Participants were selected based on the group “Patients active in the mental health care pathway” and their respective ICD codes, present in the electronic medical record of the institution. Participants diagnosed with schizophrenia were excluded from the study using the ICD-10 F20 filter, given that this disorder may be associated with complex perceptual alterations, including auditory hallucinations, which make it difficult to distinguish between psychotic symptoms and the conscious and subjective perception of tinnitus.

Participants were invited by email to answer the survey online, using a Microsoft Forms form, which consisted of the following instruments already translated and validated into Brazilian Portuguese in previous studies: tinnitus history, Tinnitus Handicap Inventory (THI)^(11)^, Visual Analog Scale (VAS)^(11)^ and Depression Anxiety and Stress Scale (DASS-21)^(12)^. The THI analyzes the degree of overall annoyance associated with tinnitus, subdivided into three domains: functional, emotional, and catastrophic. Responses are scored from zero, when tinnitus does not interfere with the patient’s life, to 100 (points or %), when the degree of annoyance is severe. The sum of the points resulting from the questions is categorized into five groups or degrees of severity: negligible (0-16%), mild (18-36%), moderate (38-56%), severe (58-76%), or catastrophic (78-100%). Regarding VAS, its indication is for measurement related to tinnitus discomfort. For analysis purposes, the following subdivision was adopted: mild for those who select scores between 0-2, moderate between 3-7, and intense from 8-10^(13)^. To track psychobehavioral aspects, the DASS-21 was used with three subscales to assess symptoms of depression, anxiety and stress presented during the last week, on a Likert-type scale from 0 (does not apply to me) to 3 (applies a lot to me, or most of the time). The scores for depression, anxiety and stress are determined by the sum of the scores of the 21 items^(12)^. All instruments used are self-reported, with no possibility of diagnostic confirmation of mental disorders or audiological evaluation of tinnitus.

The variables of interest in the study included presence and characteristics of tinnitus (frequency and intensity), tinnitus annoyance, and diagnostic screening for common mental disorders. For this analysis, the sample was divided into two groups: Study Group (SG) - patients with common mental disorders and tinnitus; Control Group (CG) - patients with common mental disorders and absence of tinnitus. The associations investigated included comparison of anxiety, depression, and stress levels between the groups; intragroup analysis of the SG on the association between the degree of tinnitus annoyance and psychobehavioral symptoms, as well as the correlation between the scale scores.

The data were collected, organized, and automatically entered into Microsoft Excel 2016 software. Incomplete responses or non-response cases were excluded from the database. The final data were analyzed using the Statistical Package for Social Sciences (SPSS), version 23. The statistical methods used for the analysis were: descriptive analysis for sample characterization, Kolmogorov-Smirnov test for normality, t-test for independent samples to compare means between groups, and Pearson’s correlation coefficient to assess associations between continuous variables. No adjustments were needed for confounding variables. A significance level of less than 5% (p<0.05) was considered for all tests.

## RESULTS

A total of 235 participants were eligible for the study, of which 153 agreed to participate. The sample was divided into two groups: 70 (45.7%) reported tinnitus and participated in the SG, and 83 (54.2%) reported no tinnitus and participated in the CG. The mean age of the SG was 37.9 years (21-67; SD = 9.9), while in the CG it was 36.6 years (21-53; SD = 7.6). The distribution of the groups by gender was 82.9% female in the SG and 81.9% in the CG, while the male distribution was 17.1% in the SG and 18.1% in the CG.

The prevalence of tinnitus in the population studied was 45.7%. This finding indicates that almost half of the participants interviewed reported experiencing tinnitus. The results presented in Table 1 indicate that most participants reported having experienced tinnitus for a period of 1 to 5 years (35.7%). The onset of tinnitus perception was predominantly gradual (70%), and the main reason related to the onset of tinnitus was stress (40%). Pulsation was absent in 70% of cases, and tinnitus was reported in both ears by most participants (45.7%).

**Table 1:** Characterization of tinnitus in the study group patients

**Variables**	**Categories**	**N**	**%**
**Duration of tinnitus perception**	3 to 6 months	8	11.4
	6 to 12 months	14	20
	1 to 5 years	25	35.7
	More than 5 years	23	32.9
**Characteristic of the onset of perception**	Sudden	21	30
	Gradual	49	70
**Probable related cause**	COVID-19	1	1.4
	Otological dermatitis	1	1.4
	Stress	28	40
	Severe flu	1	1.4
	Hearing loss	3	4.3
	Post-dental surgery	1	1.4
	Loud sound/noise	20	28.6
	Ear trauma	1	1.4
	Medication	1	1.4
	Don't know	13	18.6
**Pulse in the tinnitus**	Yes. like the heart	14	20
	Yes. different from the heart	7	10
	No	49	70
**Location of tinnitus**	Head	16	22.9
	Both ears	32	45.7
	Right ear	9	12.8
	Left ear	13	18.6
**Manifestation of tinnitus**	Constant	12	17.1
	Intermittent	58	82.9
**Change in intensity**	Yes	41	58.6
	No	29	41.4
**Type of tinnitus sound**	Whistle / cicada	33	47.1
	Hiss	4	5.7
	Click	9	12.9
	Cricket / insects	1	1.4
	Sea sound	2	2.9
	Pulse	5	7.1
	Tonal	4	5.7
	Other	10	14.3
	Whistle / cicada	2	2.9
***Pitch***	Low	2	2.9
	Medium	30	42.9
	High	33	47.1
	Very high	5	7.1
**Duration of discomfort/stress due to tinnitus in daily life over the past month**	Up to 25% of the time	37	52.9
	From 26% to 50% of the time	20	28.6
	From 51% to 75% of the time	3	4.3
	More than 75% of the time	10	14.3
**Relationship between sleep and tinnitus**	Yes	7	10
	No	14	20
	Don’t know	49	70
**Influence of stress**	Yes. it gets worse	30	42.9
	Don’t know	40	57.1
**Report of hearing disorder (self-perception)**	Yes	22	31.4
	No	48	68.6
**Physical discomfort caused by loud sounds**	Yes	26	37.1
	No	34	48.6
	Don’t know	10	14.3
**Total**		**70**	**100**

**Caption:** % = Percentage frequency; N = Absolute frequency. Source: Study data

Regarding the type, intermittent tinnitus was the most reported (82.9%), and 58.6% of participants experienced changes in sound intensity. The most common type of sound was described as a whistle or cicada-like sound (47.1%), and the predominant pitch was classified as high-pitched (47.1%). Regarding the impact of tinnitus on daily life, 52.9% of participants reported discomfort from the sound up to 25% of the time. When asked if they believed they had any hearing disorder as a result of tinnitus, 68.6% of participants reported not having noticed any hearing changes associated with the symptom, and 37.1% reported physical discomfort caused by loud sounds. Stress was mentioned as an aggravating factor by 42.9% of participants.

Regarding the degree of discomfort associated with tinnitus, measured by the VAS, moderate discomfort was reported by 75.7% of respondents. Regarding the overall degree of annoyance associated with tinnitus, measured by the THI, the “negligible” level was reported by 40% of participants. The distribution of scale values is shown in Table 2.

**Table 2:** Descriptive measures of the THI and VAS scales

**Discomfort rating - VAS**	**n**	**%**
Mild	6	8.6
Moderate	53	75.7
Intense	11	15.7
**Overall discomfort rating - THI**	**N**	**%**
Neglectable	28	40
Mild	25	35.7
Moderate	11	15.7
Severe	2	2.9
Catastrophic	4	5.7
**THI subscales**	**Mean**	**SD**
Functional	9.5	8.5
Emotional	9.4	9
Catastrophic	5.8	4.8
Overall	26.8	22.2

**Caption:** THI = Tinnitus Handicap Inventory; VAS = Visual Analog Scale; SD = Standard deviation. Source: Study data

In the analysis of the correlation between the degree of discomfort related to tinnitus, measured by the VAS, and the THI scores, a positive and moderate correlation was observed in all THI subscales. Specifically, the Pearson correlation coefficients were 0.61 for the functional subscale, 0.59 for the emotional subscale, and 0.55 for the catastrophic subscale, all with statistical significance (p < 0.001). The correlation with the total THI score was also classified as moderate, with a coefficient of 0.62 (p < 0.001).

Overall mean scores for anxiety and depression-related symptoms were higher in the group of participants with tinnitus, while stress prevailed in the group of participants without tinnitus; however, these differences did not reach statistical significance (Table 3).

**Table 3:** Descriptive measures and comparison of overall means of the DASS-21 scale between the groups with and without tinnitus

**DASS-21**	**Study group**		**Control group**		***p*-value** ^*^
	**Mean**	**SD**	**Mean**	**SD**	
Anxiety	11.4	10.4	7.9	8.3	0.099
Depression	9.7	9.9	8.4	8.9	0.648
Stress	14.5	11.6	14.5	10.1	0.101

* Independent samples t-test. Significance level adopted of 5% (p < 0.05)

**Caption:** DASS-21 = Depression Anxiety and Stress Scale; SD = Standard deviation. Source: Study data

When comparing the groups, it was observed that participants with tinnitus presented a higher prevalence of anxiety and depression scores at the mild, moderate, and extremely severe levels, compared to the control group. Furthermore, symptoms of severe and extremely severe stress were greater for participants with tinnitus, as shown in Table 4.

**Table 4:** DASS-21 scale classification between groups with and without tinnitus

**DASS-21 subscales**	**Severity**	**With Tinnitus**		**Without Tinnitus**	
		**N**	**%**	**N**	**%**
Anxiety	Normal	28	40	47	56.6
	Mild	5	7.1	5	6
	Moderate	18	25.7	15	18.1
	Severe	3	4.3	8	9.6
	Extremely Severe	16	22.9	8	9.6
Depression	Normal	41	58.6	54	65.1
	Mild	6	8.6	4	4.8
	Moderate	14	20	11	13.3
	Severe	2	2.9	12	14.5
	Extremely Severe	7	10	2	2.4
Stress	Normal	41	58.6	45	54.2
	Mild	6	8.6	14	16.9
	Moderate	8	11.4	13	15.7
	Severe	10	14.3	6	7.2
	Extremely Severe	5	7.1	5	6
**TOTAL**		**70**	**100**	**83**	**100**

**Caption:** DASS-21 = Depression Anxiety and Stress Scale; % = Percentage frequency; N = Absolute frequency. Source: Study data

Analyzing the correlation between the VAS and DASS-21 scales, a weak/negligible correlation was identified between each subscale (Anxiety, Depression, and Stress) and the VAS scale results, as shown in Table 5. In the relationship between the Functional THI subscale and the Anxiety and Stress aspects measured by the DASS-21 scale, there was a weak, albeit statistically significant, correlation. There was no significance for the other subscales, as shown in Table 5.

**Table 5:** Correlation between the THI, VAS, and DASS-21 scales

**Functional THI**	**r**	***p*-value** ^*^
DASS-21 Anxiety	0.33	**0.005**
DASS-21 Depression	0.20	0.104
DASS-21 Stress	0.29	**0.015**
**Emotional THI**		
DASS-21 Anxiety	0.17	0.164
DASS-21 Depression	0.05	0.665
DASS-21 Stress	0.19	0.120
**Catastrophic THI**		
DASS-21 Anxiety	0.10	0.415
DASS-21 Depression	0.07	0.558
DASS-21 Stress	0.09	0.475
**Overall THI**		
DASS-21 Anxiety	0.23	0.051
DASS-21 Depression	0.11	0.367
DASS-21 Stress	0.21	0.076
**VAS**		
DASS-21 Anxiety	0.15	0.214
DASS-21 Depression	0.04	0.771
DASS-21 Stress	0.08	0.505

* Significance level adopted of 5% (p < 0.05)

**Caption:** DASS-21 = Depression Anxiety and Stress Scale; THI = Tinnitus Handicap Inventory; VAS = Visual Analog Scale; r = Pearson correlation coefficient. Source: Study data

## DISCUSSION

In the present study, a tinnitus prevalence of 45.7% was estimated in the population with mental disorders followed up in primary health care, characterized predominantly by moderate discomfort. A higher frequency of elevated anxiety, depression, and stress scores was also observed among participants with tinnitus compared to those without the symptom. There was a prevalence of young adults, with the mean age similar to the findings of Lewkowski et al.^(14)^, who identified 25.9% of people with tinnitus between 35-44 years old. It should be noted that the study by Lewkowski et al.^(14)^, as well as the present study, was carried out with adults active in the labor market, which directly influences the mean age of the populations studied.

The chronicity of tinnitus correlates with its duration and influence on the quality of life of individuals. Mazurek et al.^(15)^ define chronic tinnitus as a symptom that persists for more than three months. All participants presented more than three months of tinnitus, and most participants reported changes in symptom intensity. The perception and intensity of tinnitus can vary and is associated with changes in neural activity in the auditory cortex and other brain regions over time^(16)^.

Chronic tinnitus shows different patterns of neural activity compared to recent-onset tinnitus, with increased activity in several brain areas and changes in connectivity^(16)^. A study^(17)^ that used functional magnetic resonance imaging showed that elevations in cortical activation related to tinnitus may reflect undue attention directed to the auditory domain, which may affect the perceived intensity of tinnitus, with the symptom being experienced uniquely.

In total, 47.1% of the study participants characterized tinnitus as a high-frequency whistle or cicada-like sound. Tinnitus can manifest in different types of sounds, such as pure tones or noises, and is generally perceived at frequencies corresponding to the individual’s hearing loss, when present. The frequency of tinnitus types is typically higher and can be influenced by the response of the central auditory system to the deprived auditory input, leading to changes in the tonotopic organization of the cochlea^(18)^.

Regarding the perception of sound intensity in this population, 37.1% of participants reported physical discomfort associated with loud sounds, a typical symptom of patients with auditory hypersensitivity, such as in cases of hyperacusis. In adults, hyperacusis can be associated with tinnitus, present in up to 80% of severe tinnitus cases^(19)^. This relationship is explained by the fact that tinnitus and hyperacusis involve hyperactivity and increased connectivity in the auditory-limbic-excitation-cerebellar network, indicating a complex neural network that includes auditory and non-auditory structures, with these conditions commonly overlapping^(20)^. The presence of hyperacusis can worsen the severity and impact of tinnitus over time, leading to increased complaints and greater tinnitus intensity^(21)^.

Another characteristic of tinnitus is the triggering event, worsening, or initial perception of the symptom. 40% of respondents associated tinnitus with stressful events, and 28.6% with post-exposure to intense sound or noise. Stressful events are considered significant triggering factors for the onset and/or severity of tinnitus. Furthermore, exposure to high levels of sound pressure, whether occupational, leisure or short-duration acoustic events, is a common trigger for tinnitus. The active population in the labor market is more susceptible to tinnitus, especially when there is prolonged exposure to intense sounds in headphones and/or the work environment^(22)^.

In the literature, tinnitus is commonly associated with hearing loss^(15)^. In the sample, 68.6% of participants with tinnitus reported that they do not have a diagnosis of hearing loss. Tang et al.^(23)^ highlight that tinnitus can occur without diagnosed hearing loss and its presence may be related to factors that go beyond specific damage to the inner ear, alterations in the auditory pathway or impairment of the outer hair cells, such as: somatosensory, psychobehavioral, hormonal and/or systemic causes, requiring a robust and interprofessional evaluation to identify the factors associated with the symptom.

Due to its multifactorial nature, tinnitus can trigger or worsen other symptoms or health conditions, such as sleep disorders. 10% of respondents reported a link between tinnitus and sleep. According to Feijter et al.^(24)^, sleep disorders are more frequently reported in patients with more severe self-assessed tinnitus; furthermore, tinnitus is associated with a longer sleep onset latency, reported by the patients themselves, but not with objective sleep estimates. This fact may explain the lack of perception of sleep disturbances in most of the participants.

Regarding the discomfort and/or unease caused by tinnitus, 75.7% of the study participants reported moderate discomfort, and overall discomfort ranging from negligible to mild. The factors that lead to the discomfort and unease associated with tinnitus depend on individual characteristics. Noroozian et al.^(25)^ highlight that the discomfort and unease caused by tinnitus are mainly influenced by psychological factors, duration and persistence of the symptom, and higher levels of somatization, with little influence from audiological or demographic variables. A study^(26)^ carried out with patients with shorter tinnitus experience also highlighted a predominance of negligible and mild levels of discomfort, while patients with longer tinnitus experience reported higher rates of moderate to catastrophic levels. The authors also highlight that diagnostic overlap of different conditions associated with tinnitus can worsen the perception of discomfort and unease.

The discomfort of tinnitus may be linked to its relationship with mental health conditions^(25)^. The results of this study showed a greater prevalence of mild, moderate, and extremely severe anxiety and depression scores in participants with tinnitus when compared to the group without tinnitus. In addition, symptoms of severe and extremely severe stress were greater for participants with tinnitus. However, there was no statistical significance between the variables, suggesting that although the presence of tinnitus may be related to emotional distress, a consistent difference between the groups cannot be established based solely on these measures. In the literature, studies such as that of Hackenberg et al.^(26)^ indicate that patients with tinnitus are more susceptible to mental health conditions when compared to patients without tinnitus. This fact is explained physiologically as a result of the neural connections between the perception of tinnitus and the activation of regions of the limbic system^(7)^.

Physiologically, tinnitus is associated with neural hyperactivity in auditory regions of the brain, such as the auditory cortex, and other areas involved in emotional regulation, such as the amygdala and the limbic system. Conditions such as anxiety and stress can amplify the perception of tinnitus by increasing sensitivity to internal and external stimuli, in part through activation of the hypothalamic-pituitary-adrenal (HPA) axis, resulting in a feedback loop where stress and anxiety exacerbate the perception of tinnitus, and tinnitus, in turn, intensifies the levels of these mental health conditions^(3,4)^.

In the correlation between scales, the present study also confirms the reliability in the relationship between discomfort measured by the VAS and discomfort measured by the THI with a statistically significant difference. This result is similar to the findings of Ferreira et al.^(13)^, confirming the need to use these combined scales for the characterization of tinnitus in clinical diagnosis.

However, there was no significant correlation between the scores on the THI, VAS, and DASS-21 scales, which is an important finding for clinical practice. The THI, despite having a subscale to assess the emotional impact of tinnitus, was not sufficient to measure signs and symptoms of anxiety, stress, or depression. Therefore, mental health screening is necessary to guide these patients through specific questionnaires, such as the DASS-21. Regarding the THI, the literature^(27)^ suggests that clinicians and researchers use its overall score as the main indicator of tinnitus severity, since it more robustly reflects the overall impact of the symptom, and other instruments are needed to assess aspects such as functionality and emotional factors. The study by Hamed et al.^(28)^ also used the DASS-21 for screening mental disorders in the tinnitus population. The authors^(28)^ highlight that patients with bothersome tinnitus had significantly higher scores for depression, anxiety and stress on the DASS-21 scale compared to those with non-bothersome tinnitus, suggesting the combined use of the scales.

In this context, investment in mental health programs for monitoring and guiding patients with tinnitus is crucial. Furthermore, speech-language pathologists and otolaryngologists have a role in conducting mental health assessments of these patients, providing more effective guidance and referrals. In primary health care^(8)^, systematic follow-up by the multidisciplinary health team can identify early those who need more specialized support, while referral to specialized care centers ensures multidisciplinary interventions, essential to address both the clinical aspects of tinnitus and the associated psychobehavioral impacts, thus showing the need for training professionals involved in care pathways in primary health care.

Finally, some limitations of this study should be highlighted: as it is a cross-sectional observational study, it is not possible to establish a causal relationship between tinnitus and mental disorders. Furthermore, the study was carried out in a primary health care setting within the private healthcare system, which may limit the generalizability of the findings to the general population, especially to users of the public healthcare system. It should also be considered that the diagnosis of tinnitus was made through self-report and via an online questionnaire, which may introduce bias due to memory, perception, or incomplete understanding of the questions.

## CONCLUSION

The prevalence of tinnitus was 45.75% in the sample of patients with mental disorders followed up in primary health care. It was observed that tinnitus manifested predominantly intermittently, with a high-pitched whistle or cicada-like sound, bilateral frequency, and gradual onset. An association was also found between the symptom and higher levels of anxiety, depression, and stress. Although scores were higher in the tinnitus group, not all differences were statistically significant. The occurrence of tinnitus with moderate to intense discomfort reinforces the need for integrated approaches that include specific tools to assess both the psychobehavioral impacts and the effects of tinnitus on quality of life.
