# Supplementary material for: Prevalence and characterization of tinnitus in patients with mental disorders in the context of primary health care
Source: Codas. 2026 Apr 7;38(3):e20250247. doi: 10.1590/2317-1782/e20250247en (PMC13143031; doi:10.1590/2317-1782/e20250247en)
Supplement: Translation

# Translation: Prevalência e caracterização do zumbido em pacientes com transtornos mentais no contexto da atenção primária à saúde

**DOI:** 10.1590/2317-1782/e20250247pt

**Authors:** Rubens Jonatha dos Santos Ferreira, Camila Nascimento Monteiro, Daiane Aparecida Dias, Marine Raquel Diniz da Rosa, Maria Fernanda Capoani Garcia Mondelli

**Affiliations:** 1 Programa de Pós-graduação em Fonoaudiologia, Faculdade de Odontologia de Bauru – FOB, Universidade de São Paulo – USP - Bauru (SP), Brasil.; 2 Departamento de Saúde Populacional, Hospital Sírio-Libanês - São Paulo (SP), Brasil.; 3 Programa Associado de Pós-graduação em Fonoaudiologia, Universidade Federal da Paraíba – UFPB - João Pessoa (PB), Brasil.

**Keywords:** Zumbido, Saúde Mental, Atenção Primária à Saúde, Transtornos Mentais, Prática Integral de Cuidados de Saúde

## Abstract

**Objetivo:**

Estimar a prevalência e caracterizar o zumbido de pacientes com transtornos mentais.

**Método:**

Estudo observacional realizado com pacientes inseridos na linha de cuidado de saúde mental de um serviço de atenção primária à saúde no setor suplementar. Foram utilizadas as escalas Tinnitus Handicap Inventory e Escala Visual Analógica para avaliação do zumbido, e a Depression Anxiety and Stress Scale para aspectos psicocomportamentais.

**Resultados:**

O zumbido foi relatado por quase metade da população do estudo. O estresse foi o principal fator desencadeante referido, e foram estimados maiores escores de ansiedade, depressão e estresse nos participantes com zumbido. A maioria dos participantes apresentaram desconforto moderado, e incômodo geral entre desprezível e leve.

**Conclusão:**

Foi estimada alta prevalência de zumbido com desconforto de moderado a intenso em participantes com transtornos mentais. Houve associação entre o zumbido e sintomas graves de ansiedade e estresse, destacando a importância do manejo multidisciplinar para esses pacientes.

## INTRODUÇÃO

O zumbido é um sintoma que está presente em um a cada sete adultos na atualidade, número este que está sendo ampliado pelo desencadeamento do sintoma como consequência da COVID-19^(1,2)^. Análises^(3)^ baseadas no comportamento humano apresentam modelos que enfatizam uma relação de influência entre a resposta cognitiva ao zumbido com a manifestação e o nível de incômodo associados ao sintoma.

Do ponto de vista fisiológico, algumas teorias^(4)^ indicam que a percepção do zumbido ativa áreas cerebrais relacionadas às funções executivas, como: lobo pré‐frontal, lobo parietal, córtex cingulado, ínsula, tálamo, córtex auditivo, para‐hipocampo, hipocampo e amígdala. Este fato é reforçado por pesquisas de imagens cerebrais de pessoas com zumbido^(5)^. Assim, para um manejo clínico assertivo de pacientes que relatam zumbido e dificuldades cognitivas deve-se ter compreensão desta relação, e seus possíveis efeitos aditivos de ansiedade, depressão e viés cognitivo somático^(3)^.

A associação entre o zumbido e problemas emocionais é descrito por Rosa et al.^(6)^, porém, sem caracterização do agente precursor, uma vez que a literatura descreve que portadores de zumbido podem apresentar maior tendência suicida, quadros de depressão e ansiedade, além da relação cíclica entre ansiedade, estresse, depressão e o zumbido, e seu impacto negativo para a qualidade de vida.

Além disso, a permanência do sintoma no decorrer do dia e sua interferência nas atividades sociais e de vida diária, podem influenciar no desenvolvimento de efeitos aditivos comportamentais e de saúde mental^(3)^. Uma vez que o zumbido expõe o portador a situações de estresse e distanciamento social, os comportamentos de fobia social e aspectos psicocomportamentais relacionados tendem a agravar a percepção do sintoma na relação entre o sistema límbico e circuitaria cognitiva, o que novamente remete a um ciclo constante entre estas variáveis^(7)^.

Por sua multifatorialidade e heterogeneidade, ainda não há uma explicação comum para o desencadeamento do zumbido, entretanto, sabe-se que este sintoma pode estar presente nas diferentes clínicas e níveis de atenção à saúde, dentre elas a Atenção Primária à Saúde (APS)^(8)^. A APS é um importante componente para os serviços de saúde, visto que compõe a base populacional, e oferta subsídio para o desenvolvimento de diferentes estratégias assistenciais e linhas de cuidado que resultam em melhores práticas de promoção, prevenção e reabilitação.

As linhas de cuidado em saúde mental desempenham um papel fundamental na organização, sistematização e prestação dos serviços, a fim de garantir um atendimento mais qualificado e humanizado aos pacientes, por meio da articulação e integração dos diversos níveis de atenção à saúde^(9)^. O papel da Fonoaudiologia em linhas de cuidado de saúde mental compreende desde atividades que promovam a (re)inserção dos indivíduos em meio social através do aprimoramento de habilidades comunicativas, até estratégias para minimização de agravos comportamentais associados a distúrbios da voz, audição e deglutição, que podem ser agravados em decorrência de quadros psiquiátricos, como o manejo do zumbido^(10)^.

Apesar de ser amplamente reconhecida a influência mútua entre zumbido, ansiedade, depressão e estresse, permanece a lacuna em caracterizar, de forma detalhada, os graus de incômodo e suas implicações nos desfechos emocionais e comportamentais em pacientes de saúde mental. Dessa forma, o presente estudo busca preencher esta lacuna e seus achados poderão subsidiar a qualificação das práticas clínicas e o aprimoramento de estratégias interdisciplinares de cuidado, especialmente no que se refere ao rastreamento precoce, à abordagem integral e à articulação entre os campos da saúde mental e da saúde auditiva. O estudo então se baseia na hipótese que pacientes com zumbido apresentam escores mais elevados de sintomas psicocomportamentais, quando comparados àqueles sem o sintoma. Neste cenário, o presente estudo tem por objetivo estimar a prevalência e caracterizar o zumbido de pacientes com transtornos mentais.

## MÉTODO

Este estudo configura-se como um estudo observacional e transversal, realizado com pacientes acompanhados em um programa de saúde mental na APS do setor suplementar. O projeto foi aprovado pelo Comitê de Ética em Pesquisa do Hospital Sírio-Libanês sob número 71386823.9.0000.5461. Todos os participantes assinaram o Termo de Consentimento Livre e Esclarecido.

O cálculo amostral foi baseado na população de 235 pacientes que integraram a linha de cuidado em saúde no período de junho de 2023 a junho de 2024. Considerando um desvio padrão de 1,5 pontos, nível de significância de 5% e poder do teste de 95%, a amostra foi determinada em um mínimo de 147 participantes. Considerou-se como critério de inclusão participantes maiores de 18 anos, de ambos os sexos que estavam em acompanhamento na linha de cuidado em saúde mental, possuindo um ou mais diagnósticos de transtornos mentais comuns (ansiedade- CID F40/F41; depressão- CID F32/F33; e/ou estresse- CID F43). Estes diagnósticos foram realizados previamente por médicos, com base em critérios clínicos e nos resultados das escalas utilizadas na avaliação padrão do serviço: *General Anxiety Disorder*-7 (GAD-7) e *Patient Health Questionnaire-9* (PHQ-9). Os participantes foram selecionados com base no grupo “Pacientes ativos na linha de cuidado de saúde mental” e respectivos CIDs, presentes no prontuário eletrônico da instituição. Foram excluídos do estudo participantes com diagnóstico de esquizofrenia, por meio de filtragem do CID F20, tendo em vista que esse transtorno pode estar associado a alterações perceptivas complexas, incluindo alucinações auditivas, que dificultam a distinção entre sintomas psicóticos e a percepção consciente e subjetiva do zumbido.

Os participantes foram convidados por e-mail para responder a pesquisa de forma online, por meio de um formulário do Microsoft Forms, composto pelos seguintes instrumentos já traduzidos e validados para o português brasileiro em estudos anteriores: anamnese do zumbido, *Tinnitus Handicap Inventory* (THI)^(11)^, Escala Visual Analógica (EVA)^(11)^ e *Depression Anxiety and Stress Scale* (DASS-21)^(12)^. O THI analisa o grau de incômodo geral associado ao zumbido, sendo subdivido em três domínios: funcional, emocional e catastrófico. As respostas são pontuadas de zero, quando o zumbido não interfere na vida do paciente, até 100 (pontos ou %), quando o grau de incômodo é grave. A somatória dos pontos resultantes das questões é categorizada em cinco grupos ou graus de gravidade: desprezível (0-16%), leve (18-36%), moderado (38-56%), severo (58-76%) ou catastrófico (78-100%). Quanto a EVA, sua indicação é para mensuração relacionada ao desconforto com o zumbido. Para efeitos de análise foi adotada a subdivisão: Leve para aqueles que selecionarem pontuações entre 0-2, moderado entre 3-7, e intenso de 8-10^(13)^. Para rastreio de aspectos psicocomportamentais foi utilizada a DASS-21 com três subescalas para avaliar sintomas de depressão, ansiedade e estresse apresentados durante a última semana, em uma escala do tipo Likert de 0 (não se aplica a mim) a 3 (aplica-se muito a mim, ou a maior parte do tempo). As pontuações para depressão, ansiedade e estresse são determinadas pela soma dos escores dos 21 itens^(12)^. Todos os instrumentos utilizados são autorreferidos, sem possibilidade de confirmação diagnóstica de transtornos mentais ou avaliação audiológica do zumbido.

As variáveis de interesse do estudo foram: presença e características do zumbido (frequência e intensidade), incômodo do zumbido e rastreio diagnóstico de transtornos mentais comuns. Para esta análise, a amostra foi dividida em dois grupos: Grupo de Estudo (GE) pacientes com transtornos mentais comuns e zumbido; Grupo Controle (GC) pacientes com transtornos mentais comuns e ausência de zumbido. As associações investigadas foram: comparação dos níveis de ansiedade, depressão e estresse entre os grupos; análise intragrupo do GE sobre a associação entre o grau de desconforto do zumbido e sintomas psicocomportamentais, além da correlação entre os escores das escalas.

Os dados foram coletados, organizados e imputados no software Microsoft Excel 2016 de forma automática. Respostas incompletas ou os casos de não resposta foram excluídos da base de dados. Os dados finais foram analisados no *Statistical Package for Social Sciences* (SPSS), versão 23. Para a análise, foram utilizados como métodos estatísticos: análise descritiva para caracterização da amostra, Teste de *Kolmogorov-Smirnov* para normalidade, Teste T para amostras independentes para comparar médias entre grupos e coeficiente de correlação de Pearson para avaliar associações entre variáveis contínuas. Não houve necessidade de ajustes para variáveis de confusão. Para todos os testes foi considerado o nível de significância inferior à 5% (p<0,05).

## RESULTADOS

Foram elegíveis para a pesquisa 235 participantes, dos quais 153 aceitaram participar do estudo. A amostra foi dividida em duas partes, sendo 70 (45,7%) deles com zumbido referido, participantes do GE, e 83 (54,2%) respondentes sem zumbido, participantes GC. A idade média do GE foi 37,9 anos (21-67; DP = 9,9), enquanto no GC foi de 36,6 anos (21-53; DP= 7,6). A distribuição dos grupos por sexo foi de 82,9% do sexo feminino no GE e 81,9% no GC, enquanto do sexo masculino foi de 17,1% no GE e 18,1% no GC.

A prevalência do zumbido na população estudada foi de 45,7%. Esse achado indica que praticamente um em cada dois participantes entrevistados relatou presença de zumbido. Os resultados apresentados na Tabela 1 indicam que a maioria dos participantes relatou ter percebido o zumbido por um período de 1 a 5 anos (35,7%). O início da percepção do zumbido foi predominante de forma gradual (70%), e o principal motivo relacionado ao surgimento do zumbido foi o estresse (40%). A pulsação no zumbido foi ausente em 70% dos casos, e o zumbido localizou-se em ambas as orelhas na maioria dos participantes (45,7%).

**Tabela 1:** Caracterização do zumbido dos pacientes do grupo de estudo

**Variáveis**	**Categorias**	**N**	**%**
**Tempo de percepção do zumbido**	De 3 a 6 meses	8	11,4
	De 6 a 12 meses	14	20
	De 1 a 5 anos	25	35,7
	Mais de 5 anos	23	32,9
**Característica do início da percepção**	Súbita	21	30
	Gradual	49	70
**Provável motivo relacionado**	COVID-19	1	1,4
	Dermatite otológica	1	1,4
	Estresse	28	40
	Gripe forte	1	1,4
	Perda auditiva	3	4,3
	Pós-cirurgia dentária	1	1,4
	Som/ruído intenso	20	28,6
	Trauma no ouvido	1	1,4
	Uso de medicamentos	1	1,4
	Não sabe	13	18,6
**Pulsação no zumbido**	Sim, como o coração	14	20
	Sim, diferente do coração	7	10
	Não	49	70
**Local do zumbido**	Cabeça	16	22,9
	Ambas as orelhas	32	45,7
	Orelha direita	9	12,8
	Orelha esquerda	13	18,6
**Manifestação do zumbido**	Constante	12	17,1
	Intermitente	58	82,9
**Mudança de intensidade**	Sim	41	58,6
	Não	29	41,4
**Tipo de som do zumbido**	Apito / cigarra	33	47,1
	Assobio	4	5,7
	Chiado	9	12,9
	Click	1	1,4
	Grilo / insetos	2	2,9
	Barulho do mar	5	7,1
	Pulsação	4	5,7
	Tonal	10	14,3
	Outros	2	2,9
***Pitch***	Grave	2	2,9
	Médio	30	42,9
	Agudo	33	47,1
	Muito agudo	5	7,1
**Tempo de incômodo/estresse pelo zumbido no dia a dia no último mês**	Até 25% do tempo	37	52,9
	De 26% a 50% do tempo	20	28,6
	De 51% a 75% do tempo	3	4,3
	Mais de 75% do tempo	10	14,3
**Relação do sono com o zumbido**	Sim	7	10
	Não	14	20
	Não sabe	49	70
**Influência do estresse**	Sim, piora	30	42,9
	Não sabe	40	57,1
**Relato de problemas de audição (autopercepção)**	Sim	22	31,4
	Não	48	68,6
**Desconforto físico ocasionado por sons intensos**	Sim	26	37,1
	Não	34	48,6
	Não sabe	10	14,3
**Total**		**70**	**100**

**Legenda:** % = Frequência percentual; N = Frequência absoluta. Fonte: Dados do estudo

Em termos de manifestação, o zumbido intermitente foi o mais relatado (82,9%), e 58,6% dos participantes experimentaram mudanças na intensidade do som. O tipo de som mais comum foi descrito como apito ou cigarra (47,1%), e o *pitch* predominante foi classificado como agudo (47,1%). Quanto ao impacto do zumbido no cotidiano, 52,9% dos participantes relataram incômodo pelo som em até 25% do tempo. Quando questionados se acreditavam apresentar algum problema auditivo em decorrência do zumbido, 68,6% dos participantes relataram não perceber alterações auditivas associadas ao sintoma. 37,1% relataram desconforto físico ocasionado por sons intensos. O estresse foi apontado como fator agravante por 42,9% dos participantes.

Quanto ao grau de desconforto associado ao zumbido, mensurado pela EVA, foi observada predominância do desconforto moderado em 75,7% dos respondentes. Em relação aos graus de incômodo geral associado ao zumbido, medidos pelo THI, predominou o grau “desprezível” apontado por 40% dos participantes. A distribuição dos valores das escalas pode ser observada na Tabela 2.

**Tabela 2:** Medidas descritivas das escalas THI e EVA

**Classificação desconforto- EVA**	**n**	**%**
Leve	6	8,6
Moderado	53	75,7
Intenso	11	15,7
**Classificação geral incômodo- THI**	**N**	**%**
Desprezível	28	40
Leve	25	35,7
Moderado	11	15,7
Severo	2	2,9
Catastrófico	4	5,7
**Subescalas THI**	**Média**	**DP**
Funcional	9,5	8,5
Emocional	9,4	9
Catastrófico	5,8	4,8
Geral	26,8	22,2

**Legenda:** THI = *Tinnitus Handicap Inventory*; EVA = Escala Visual Analógica; DP = Desvio padrão. Fonte: Dados do estudo

Na análise da correlação entre o grau de desconforto relacionado ao zumbido, mensurado pela EVA, e os escores do THI, observou-se correlação positiva e moderada em todas as subescalas do THI. Especificamente, os coeficientes de correlação de Pearson foram de 0,61 para a subescala funcional, 0,59 para a emocional e 0,55 para a catastrófica, todos com significância estatística (p < 0,001). A correlação com o escore total do THI também foi classificada como moderada, com coeficiente de 0,62 (p < 0,001).

Os escores médios gerais dos sintomas relacionados a ansiedade e depressão foram superiores no grupo de participantes com zumbido, enquanto o estresse prevaleceu no grupo de participantes sem zumbido, entretanto, essas diferenças não alcançaram significância estatística (Tabela 3).

**Tabela 3:** Medidas descritivas e comparação das médias gerais da escala DASS-21 entre os grupos com e sem zumbido

**DASS-21**	**Grupo de estudo**		**Grupo controle**		***p*-valor** ^*^
	**Média**	**DP**	**Média**	**DP**	
Ansiedade	11,4	10,4	7,9	8,3	0,099
Depressão	9,7	9,9	8,4	8,9	0,648
Estresse	14,5	11,6	14,5	10,1	0,101

* Teste t para amostras independentes. Nível de significância adotado de 5% (*p* < 0,05)

**Legenda:** DASS-21 = Depression Anxiety and Stress Scale; DP = Desvio padrão. Fonte: Dados do estudo

Ao comparar os grupos, observou-se que os participantes com zumbido apresentaram maior predominância de escores de ansiedade e depressão nos níveis leve, moderado e extremamente grave, em relação ao grupo controle. Além disso, os sintomas de estresse grave e extremamente grave foram superiores para os participantes com zumbido, conforme apresenta a Tabela 4.

**Tabela 4:** Classificação da escala DASS-21 entre os grupos com e sem zumbido

**Subescalas DASS-21**	**Severidade**	**Com Zumbido**		**Sem Zumbido**	
		**N**	**%**	**N**	**%**
Ansiedade	Normal	28	40	47	56,6
	Leve	5	7,1	5	6
	Moderado	18	25,7	15	18,1
	Grave	3	4,3	8	9,6
	Extremamente Grave	16	22,9	8	9,6
Depressão	Normal	41	58,6	54	65,1
	Leve	6	8,6	4	4,8
	Moderado	14	20	11	13,3
	Grave	2	2,9	12	14,5
	Extremamente Grave	7	10	2	2,4
Estresse	Normal	41	58,6	45	54,2
	Leve	6	8,6	14	16,9
	Moderado	8	11,4	13	15,7
	Grave	10	14,3	6	7,2
	Extremamente Grave	5	7,1	5	6
**TOTAL**		**70**	**100**	**83**	**100**

**Legenda:** DASS-21 = Depression Anxiety and Stress Scale; % = Frequência percentual; N = Frequência absoluta. Fonte: Dados do estudo

Analisando a correlação entre as escalas EVA e DASS-21, identificou-se correlação fraca/desprezível entre cada sub escala (Ansiedade, Depressão e Estresse) e os resultados da escala EVA, conforme apresentado na Tabela 5. Na relação entre a subescala THI Funcional e os aspectos de Ansiedade e Estresse medidos pela escala DASS-21 houve uma correlação fraca, ainda que estatisticamente significativa. Não houve significância para as demais subescalas, conforme apresentado na Tabela 5.

**Tabela 5:** Correlação entre as escalas THI, EVA e DASS-21

**THI Funcional**	**r**	***p*-valor** ^*^
DASS-21 Ansiedade	0,33	**0,005**
DASS-21 Depressão	0,20	0,104
DASS-21 Estresse	0,29	**0,015**
**THI Emocional**		
DASS-21 Ansiedade	0,17	0,164
DASS-21 Depressão	0,05	0,665
DASS-21 Estresse	0,19	0,120
**THI Catastrófico**		
DASS-21 Ansiedade	0,10	0,415
DASS-21 Depressão	0,07	0,558
DASS-21 Estresse	0,09	0,475
**THI Geral**		
DASS-21 Ansiedade	0,23	0,051
DASS-21 Depressão	0,11	0,367
DASS-21 Estresse	0,21	0,076
**EVA**		
DASS-21 Ansiedade	0,15	0,214
DASS-21 Depressão	0,04	0,771
DASS-21 Estresse	0,08	0,505

* Nível de significância adotado de 5% (*p* < 0,05)

**Legenda:** DASS-21 = Depression Anxiety and Stress Scale; THI = Tinnitus Handicap Inventory; EVA = Escala Visual Analógica; r = coeficiente de correlação de Pearson. Fonte: Dados do estudo

## DISCUSSÃO

No presente estudo, estimou-se uma prevalência de zumbido de 45,7% na população com transtornos mentais acompanhada na APS, caracterizada predominantemente por desconforto moderado. Observou-se ainda maior frequência de escores elevados de ansiedade, depressão e estresse entre participantes com zumbido, em comparação àqueles sem o sintoma. Houve prevalência de adultos-jovens, sendo a idade média do estudo semelhante aos achados de Lewkowski et al.^(14)^ que identificaram 25,9% das pessoas com zumbido entre 35-44 anos. Ressalta-se que o estudo de Lewkowski et al.^(14)^, assim como o presente estudo, foi realizado com adultos ativos no mercado de trabalho, o que influencia diretamente na média de idade das populações estudadas.

A cronicidade do zumbido está correlacionada com sua duração e influência na qualidade de vida dos indivíduos. Mazurek et al.^(15)^ definem como zumbido crônico, o sintoma que persiste por mais de três meses. Todos os participantes apresentaram mais de três meses de zumbido, e a maior parte dos participantes relata haver mudança de intensidade do sintoma. A percepção e intensidade do zumbido pode variar e está associada a mudanças na atividade neural no córtex auditivo e outras regiões do cérebro ao longo do tempo^(16)^.

O zumbido crônico mostra diferentes padrões de atividade neural em comparação ao zumbido de início recente, com aumento da atividade em várias áreas do cérebro e mudanças na conectividade^(16)^. Um estudo^(17)^ que utilizou ressonância magnética funcional, apontou que elevações na ativação cortical relacionadas ao zumbido podem refletir atenção indevida direcionada ao domínio auditivo, o que pode afetar a intensidade percebida do zumbido, sendo o sintoma experienciado de forma única.

No total, 47,1% dos participantes do estudo caracterizaram a manifestação sonora do zumbido como som de apito ou cigarra de frequência aguda. O zumbido pode se manifestar em diferentes tipos de sons, como tons puros ou ruídos, sendo geralmente percebido em frequências correspondentes à perda auditiva do indivíduo, quando existente. A frequência dos tipos de zumbido é tipicamente mais alta e pode ser influenciada pela resposta do sistema auditivo central à entrada auditiva privada, levando a mudanças na organização tonotópica da cóclea^(18)^.

No que se refere a percepção de intensidade sonora nesta população, 37,1% dos participantes relataram desconforto físico associado a sons intensos, um sintoma típico de pacientes com hipersensibilidade auditiva, como nos quadros de hiperacusia. Em adultos, a hiperacusia pode estar associada ao zumbido, presente em até 80% dos casos graves de zumbido^(19)^. Esta relação se explica pois o zumbido e a hiperacusia envolvem hiperatividade e conectividade aumentada na rede auditiva-límbica-excitação-cerebelar, indicando uma rede neural complexa que inclui estruturas auditivas e não auditivas, sendo comum a sobreposição destes quadros^(20)^. A presença de hiperacusia pode piorar a gravidade e o sofrimento do zumbido ao longo do tempo, levando ao aumento das queixas e à maior intensidade do zumbido^(21)^.

Outro fator de individualidade do zumbido, é o evento de desencadeamento, agravamento ou percepção inicial do sintoma. 40% dos entrevistados associaram o zumbido a eventos estressantes e 28,6% pós exposição a som ou ruído intenso. Eventos estressantes são considerados fatores desencadeantes significativos para o início e/ou gravidade do zumbido. Além disso, a exposição a níveis elevados de pressão sonora, sejam eles ocupacionais, de lazer ou eventos acústicos de curta duração, é um fator desencadeante comum para o zumbido. A população ativa no mercado de trabalho está mais suscetível ao zumbido, principalmente quando há exposição prolongada a sons intensos nos fones de ouvido e/ou cenário laboral^(22)^.

Na literatura o zumbido é comumente associado a perdas auditivas^(15)^. Na amostra, 68,6% dos participantes com zumbido relataram que não possuem diagnósticos de problemas auditivos. Tang et al.^(23)^ destacam que o zumbido pode ocorrer sem perda auditiva diagnosticada e sua presença pode estar relacionada a fatores que vão além dos danos específicos na orelha interna, alterações da via auditiva ou comprometimento das células ciliadas externas, como: causas somatossensoriais, psicocomportamentais, alterações hormonais e/ou sistêmicas, sendo necessária uma avaliação robusta e interprofissionais para identificar os fatores associados ao sintoma.

Devido à sua multifatorialidade o zumbido pode desencadear ou agravar outros sintomas ou quadros de saúde, como os distúrbios do sono. 10% dos entrevistados relataram haver uma ligação entre o zumbido e o sono. De acordo com Feijter et al.^(24)^ os distúrbios do sono são mais relatados em pacientes com maior gravidade de zumbido autoavaliada, além disso, o zumbido está associado a uma latência maior para o início do sono, relatada pelos próprios pacientes, mas não a estimativas objetivas do sono. Este fato pode explicar a ausência da percepção de alterações do sono na maior parte da população da pesquisa.

Em relação ao incômodo e/ou desconforto gerado pelo zumbido, foi estimado que 75,7% dos participantes do estudo apresentaram desconforto moderado, e incômodo geral entre desprezível e leve. Os fatores que levam ao incômodo e desconforto associados ao zumbido são dependentes de características individuais. Noroozian et al.^(25)^ destacam que o desconforto e incômodo causados pelo zumbido são influenciados principalmente por fatores psicológicos, tempo de duração e permanência do sintoma e níveis mais altos de somatização, com pouca influência de variáveis ​​audiológicas ou demográficas. Um estudo^(26)^ realizado com pacientes com menor tempo de convívio com zumbido também destacou predominância nos graus desprezível e leve, enquanto pacientes com maior tempo de manifestação do zumbido reportaram maiores índices de grau moderado a catastrófico. Os autores também destacam que a sobreposição diagnóstica de diferentes quadros associados ao zumbido pode agravar a percepção do incômodo e desconforto.

O incômodo do zumbido pode estar ligado à sua relação com quadros de saúde mental^(25)^. Os resultados deste estudo demonstraram maior predominância dos escores de ansiedade e depressão leve, moderada e extremamente grave em participantes com zumbido, quando comparado ao grupo sem zumbido. Além disso, os sintomas de estresse grave e extremamente grave foram superiores para os participantes com zumbido. Entretanto, não houve significância estatística entre as variáveis e sugere que, embora a presença do zumbido possa estar relacionada a sofrimento emocional, não se pode afirmar uma diferença consistente entre os grupos com base apenas nestas medidas. Na literatura, estudos como o de Hackenberg et al.^(26)^apontam que pacientes com zumbido são mais susceptíveis à quadros de saúde mental, quando comparado a pessoas sem o zumbido. Este fato é explicado fisiologicamente em decorrência das ligações neurais entre a percepção do zumbido e ativação de regiões do sistema límbico^(7)^.

Fisiologicamente, o zumbido está associado à hiperatividade neural nas regiões auditivas do cérebro, como o córtex auditivo, e a outras áreas envolvidas na regulação emocional, como a amígdala e o sistema límbico. Quadros como a ansiedade e estresse podem amplificar a percepção do zumbido ao aumentar a sensibilidade a estímulos internos e externos, em parte por meio da ativação do eixo hipotálamo-hipófise-adrenal (HPA), o que resulta em um ciclo de retroalimentação, em que o estresse e a ansiedade exacerbam a percepção do zumbido, e o zumbido, por sua vez, intensifica os níveis destes quadros de saúde mental^(3,4)^.

Na correlação entre escalas, a presente pesquisa ainda confirma a confiabilidade na relação entre o desconforto mensurado pela EVA e incômodo mensurado pelo THI com diferença estatisticamente significativa. Este resultado foi similar aos achados de Ferreira et al.^(13)^, confirmando a necessidade de utilização destas escalas combinadas para a caracterização do zumbido no diagnóstico clínico.

Entretanto, não houve correlação significativa entre os valores de escore das escalas THI, EVA e DASS-21, sendo este um dado importante para a prática clínica. O THI, apesar de ter uma subescala para avaliação do impacto emocional do zumbido, não foi suficiente para mensurar sinais e sintomas de ansiedade, estresse ou depressão, sendo necessário um rastreio de saúde mental para direcionamento desses pacientes através de questionários específicos, como o DASS-21. Sobre o THI, a literatura^(27)^ sugere que clínicos e pesquisadores utilizem sua pontuação geral como principal indicador da gravidade do zumbido, visto que reflete de forma mais robusta o impacto global do sintoma, sendo necessária a utilização de outros instrumentos para avaliar aspectos como funcionalidade e fatores emocionais. O estudo de Hamed et al.^(28)^ também utilizou a DASS-21 para rastreio de transtornos mentais na população com zumbido. Os autores^(28)^ destacam que pacientes com zumbido incômodo apresentaram pontuações significativamente mais altas para depressão, ansiedade e estresse na escala DASS-21 em comparação com aqueles com zumbido não incômodo, sugerindo o uso combinado das escalas.

Neste cenário, é fundamental o investimento em programas de saúde mental para acompanhamento e direcionamento dos pacientes com zumbido. Além disso, cabe aos profissionais da Fonoaudiologia e Otorrinolaringologia a realização de rastreios de saúde mental nesses pacientes, proporcionando orientações e encaminhamentos mais assertivos. Na APS^(8)^, o acompanhamento sistemático pela equipe multiprofissional de saúde poderá identificar precocemente aqueles que necessitam de suporte mais especializado, enquanto o encaminhamento para centros de cuidado especializado garante intervenções multidisciplinares, essenciais para abordar tanto os aspectos clínicos do zumbido quanto os impactos psicocomportamentais associados, mostrando assim a necessidade da capacitação dos profissionais envolvidos em linhas de cuidado na APS.

Por fim, destacam-se algumas limitações deste estudo: por se tratar de um estudo observacional transversal, não é possível estabelecer uma relação de causalidade entre o zumbido e transtornos mentais. Além disso, o estudo foi realizado em cenário de APS na saúde suplementar, podendo limitar a generalização dos achados para a população em geral, especialmente para usuários do Sistema Único de Saúde. Deve-se considerar também que o diagnóstico do zumbido foi feito por autorrelato e por meio de questionário virtual, o que pode introduzir viés de memória, percepção ou não entendimento total das questões.

## CONCLUSÃO

A prevalência do zumbido foi de 45,75% na amostra de pacientes com transtornos mentais acompanhados na atenção primária à saúde. Observou-se que o zumbido apresentou, predominantemente, manifestação intermitente, de *pitch* agudo e tipo apito ou cigarra, com frequência bilateral e início gradual. Foi evidenciado ainda associação entre o sintoma e maiores índices de ansiedade, depressão e estresse. Embora os escores tenham sido maiores no grupo com zumbido, nem todas as diferenças foram estatisticamente significativas. A ocorrência de zumbido com desconforto moderado a intenso reforça a necessidade de abordagens integradas, que incluam ferramentas específicas para avaliar tanto os impactos psicocomportamentais quanto os efeitos do zumbido na qualidade de vida.
